# The Role of Care

**DOI:** 10.1080/11287462.2021.2011007

**Published:** 2022-02-04

**Authors:** Christine Overall

**Affiliations:** Queen’s University, Kingston, Canada

**Keywords:** Rosemarie Tong, care, justice, global bioethics, indigenous peoples

## Abstract

“The Role of Care” is a commentary on “Towards a Feminist Global Ethics,” by Rosemarie Tong.

I have great respect for Rosemarie Tong’s work, for her personal history of contributions to feminist ethics and bioethics, and for her wealth of knowledge about global bioethics. “Towards a Feminist Global Ethics” (Tong 2021) offers a powerful discussion, bringing much of the history of feminist ethics to bear upon the challenge of defining an approach to feminist global bioethics. Because I do not have space to comment on all aspects of the paper, I shall concentrate upon the central theme, Dr. Tong’s deployment of the concept of care, and her view that it provides a “universalism” that is, on its own, essential to global bioethics.

She writes, “[T]here is room in global bioethics for a certain type of non-imperialistic, non-colonial universalism that is present in some formulations of *feminist* global bioethics,” (Tong, 6, her emphasis) and she proposes a “future, care-based feminist global bioethics” (32). Tong frames the other side of the debate about frameworks for global bioethics as being concerned with justice and rights. She writes, “Like Virginia Held, I think care has a priority over justice” (31). But while there is a good case to be made for the role of care in feminist global bioethics, I am not convinced that it should have priority over justice.

The justice framework, broadly and fairly construed, concerns itself with oppression, marginalization, exploitation, and misappropriation. These include injustices of sex/gender, race, class, age, sexuality, and disability. There are also the monstrous injustices committed against indigenous peoples all over the world – injustices against their lands, their cultures, and their bodies. There is climate injustice and the arrogation of planetary resources, along with the injustices causing and resulting from global catastrophes such as the Covid pandemic. And, I would add, there is the injustice of the exploitation and appropriation of non-human animal bodies.

Of course Dr. Tong knows about and acknowledges these enormous inequities. The problem is that if global bioethics is framed primarily in terms of care, then immoral actions, policies, and social conditions appear to be caused by and to express a *lack* of care. But what makes certain actions, policies, and social conditions immoral is far more, and more complex, than a lack of care. They are the manifestation and result of broad social ideologies and institutions such as religious hegemony, misogyny, racism, ageism, ableism, and heterosexism. They are the outcome of the forces of nationalism, colonialism, imperialism, capitalism, and the choices of colonizers/occupiers, capitalists, and developed-world consumers. Perhaps most of all, they are the consequence of global inequalities: the unjust distribution of resources and advantages, liabilities and burdens, obligations and rights.

Looking at the lengthy list provided by Dr. Tong of issues that global bioethics deals with (4-5), it is hard to see how care can resolve them. This inadequacy arises from three factors: First, many of these issues involve the power differentials of race, sex/gender, sexual identity, age, class, and ability. Second, the “sensitivity to feelings” and the “love, affection, compassion, or sympathy” (23) that are mandated by an ethics of care are the emotions and attitudes of individual persons, and they provide no clear means or methods of resolving moral problems related to identity groups, communities, nations, or the planet. Certainly these emotions and attitudes can be learned, and they *may* have played some role in the reduction of persecution of LGBTQ+ people. Still, they are not enough to defeat oppression. Third, it is unclear how the “cultivat[ion of] traditional feminine virtues” and the commitment to a “subsistence perspective” (28) – while clearly attainable by individuals, families, and small communities – can be adapted to resolving “patenting disputes over pharmaceutical and medical equipment; genetic material controversies … ; the buying and selling of reproductive material … ; ‘medical tourism’ … ; trade in human organs and tissues; and public health emergencies” (4-5) – that is, the kinds of issues that Dr. Tong regards as representative of global bioethical challenges.

Dr. Tong writes, “care is a more fundamental moral value or practice than justice” (1). Care certainly has a role to play in interactions among individuals, and perhaps also in the *motivations* of moral actors and the *goals* of moral actions and policies. But what is needed in the paper are examples of the *use* of a care framework to resolve the issues in global bioethics that Dr. Tong has identified, and a description of the methods or tools by such resolutions might be achieved. I am not asking for an “ethics that ‘tells us what to do’” (Fiona Robinson, quoted on p. 24). But I am asking for at least some hints about how care, as a “universalism,” contributes to mitigating such phenomena as diverse as ableism, the climate crisis, and the imperialist appropriation of the lands and cultures of Indigenous peoples. In the absence of such hints, while I am open to the idea that care has a role to play in feminist global bioethics, I am not yet convinced that it is as powerful as Dr. Tong believes, and that it can by itself handle the varieties of oppression and inequality to which I’ve gestured here.

Perhaps, then, in order to develop a feminist global bioethics, what is needed is an *intersectional* approach to the values of care and justice. Given that it is possible and desirable to take an intersectional approach to identity characteristics like race and sex/gender, maybe it’s also possible to create an intersectional approach to these two significant ethical standards, justice and care, even though identities and ethical standards belong to two different categories. How might this be done? I don’t have an answer, but I do have a real-world example, which I think exemplifies the pragmatic intersection of care and justice.

In the summer of 2021 in Canada, hundreds of unmarked graves were rediscovered, of Indigenous children who had died at government- and church-sponsored residential schools. The children were taken to the schools in the nineteenth and twentieth centuries, often against their parents’ will, with the express intent of enculturating them in colonial values and practices. The children were often badly treated, and their health suffered, frequently at the cost of their lives (Staff, 2021).

In response to these rediscoveries, there have been demands from Indigenous peoples and their allies to take down monuments to the founders and supporters of the schools, to compel the Vatican to apologize for the role in the schools of the Catholic Church, and to change the Canadian Oath of Citizenship to include references to Indigenous peoples and treaty rights. These responses to the rediscoveries point to and demand reparations for the huge injustices inflicted on the Indigenous peoples of Turtle Island by colonizers/occupiers.

On many evenings that same summer, I heard through my window some beautiful singing by a group of Indigenous women. They and their male colleagues were peacefully occupying a small square in a park near my home. The women’s music was haunting, mournful, sometimes exultant. One day I went to the square where the women were tending a sacred fire. I said to one of the women, “I love your singing.” The woman thanked me. Then I said, “I know you’re not singing for me, but I want you to know I think it’s beautiful.” The woman replied, “But we *are* singing for you. We’re singing to heal everybody.”

I suggest that in these two different sets of responses we see *both* a concern for justice, manifested in the reparative demands made by Indigenous people, *and* a concern for care, manifested by the Indigenous women who sing to heal everyone. These actions are compatible with each other; indeed, they reinforce each other. Thus, responding to the unjust and uncaring treatment of the Indigenous children can and does mean both seeking justice and providing care. This example shows that both ethical standards, care and justice, may serve as motivation, method, and goal in the struggle against oppression.
